# Any versus long-term prescribing of high risk medications in older people using 2012 Beers Criteria: results from three cross-sectional samples of primary care records for 2003/4, 2007/8 and 2011/12

**DOI:** 10.1186/s12877-015-0143-8

**Published:** 2015-11-05

**Authors:** Alessandro Ble, Jane A. H. Masoli, Heather E. Barry, Rachel E. Winder, Behrooz Tavakoly, William E. Henley, George A. Kuchel, Jose M. Valderas, David Melzer, Suzanne H. Richards

**Affiliations:** Epidemiology and Public Health, Institute of Biomedical and Clinical Science, University of Exeter Medical School, Barrack Road, Exeter, EX2 5DW UK; National Institute for Health Research (NIHR)‘School for Public Health Research, ᅟ, UK; Healthcare for Older People, Royal Devon and Exeter NHS Foundation Trust, Barrack Rd, Exeter, EX2 5DW UK; Primary Care, Institute of Health Research, University of Exeter Medical School, Smeall Building, St Luke’s Campus, Exeter, EX1 2LU UK; Epidemiology and Public Health, Institute of Biomedical and Clinical Science, University of Exeter Medical School, Smeall Building, St Luke’s Campus, Exeter, EX1 2LU UK; Health Statistics, Institute of Health Research, University of Exeter Medical School, College House, St Luke’s Campus, Exeter, EX1 2LU UK; UConn Center on Aging, University of Connecticut Health Center, 263 Farmington Avenue, Farmington, CT 06030-5215 USA; Health Services & Policy Research, Institute of Health Research, University of Exeter Medical School, Smeall Building, St Luke’s Campus, Exeter, EX1 2LU UK; Primary Care Research Group, Institute of Health Research, University of Exeter Medical School, Smeall Building, St Luke’s Campus, Exeter, EX1 2LU UK

**Keywords:** Polypharmacy, Older people, High risky medications, Potentially inappropriate prescribing, General practice, Electronic medical records, Observational study, Beers criteria, Family medicine

## Abstract

**Background:**

High risk medications are commonly prescribed to older US patients. Currently, less is known about high risk medication prescribing in other Western Countries, including the UK. We measured trends and correlates of high risk medication prescribing in a subset of the older UK population (community/institutionalized) to inform harm minimization efforts.

**Methods:**

Three cross-sectional samples from primary care electronic clinical records (UK Clinical Practice Research Datalink, CPRD) in fiscal years 2003/04, 2007/08 and 2011/12 were taken. This yielded a sample of 13,900 people aged 65 years or over from 504 UK general practices. High risk medications were defined by 2012 Beers Criteria adapted for the UK. Using descriptive statistical methods and regression modelling, prevalence of ‘any’ (drugs prescribed at least once per year) and ‘long-term’ (drugs prescribed all quarters of year) high risk medication prescribing and correlates were determined.

**Results:**

While polypharmacy rates have risen sharply, high risk medication prevalence has remained stable across a decade. A third of older (65+) people are exposed to high risk medications, but only half of the total prevalence was long-term (any = 38.4 % [95 % CI: 36.3, 40.5]; long-term = 17.4 % [15.9, 19.9] in 2011/12). Long-term but not any high risk medication exposure was associated with older ages (85 years or over). Women and people with higher polypharmacy burden were at greater risk of exposure; lower socio-economic status was not associated. Ten drugs/drug classes accounted for most of high risk medication prescribing in 2011/12.

**Conclusions:**

High risk medication prescribing has not increased over time against a background of increasing polypharmacy in the UK. Half of patients receiving high risk medications do so for less than a year. Reducing or optimising the use of a limited number of drugs could dramatically reduce high risk medications in older people. Further research is needed to investigate why the oldest old and women are at greater risk. Interventions to reduce high risk medications may need to target shorter and long-term use separately.

**Electronic supplementary material:**

The online version of this article (doi:10.1186/s12877-015-0143-8) contains supplementary material, which is available to authorized users.

## Background

Polypharmacy, the use of multiple drugs by the same individual [1, 2], is increasingly common in older people in both the United States (US) [3] and United Kingdom (UK) [4].

Over the past decade, the average number of prescribed items per person per year in England rose from 12.4 in 2002 to 18.7 in 2012; patients aged 60 years or over received half of all prescribed items [4]. Any medicine has the potential for benefits as well as risks [5, 6], but older people are at greater risk of adverse reactions because of age-associated changes affecting pharmacokinetics and pharmacodynamics [7].

Various tools [5, 8], including the START/STOPP toolkit [9] and the Beers Criteria [10–12], have been created to help clinicians to minimise risk by avoiding the use of drugs with a higher risk-to-benefit ratio in older people. Although historically, it has been proposed that Beers criteria have weaknesses when used in European clinical settings [13], recently it has shown the Beers criteria to be more sensitive and complementary to tools developed for use in Europe (e.g. STOPP) [13, 14]. The Beers Criteria is endorsed by the American Geriatrics Society and their recommendations have recently been updated through systematic review of the evidence base. Beers lists drugs (or drug classes) with potentially harmful effects in the older population, including those consistently associated with poor patient outcomes (adverse drug reactions, hospitalisation, and mortality) [12]. To develop the 2012 update, a multidisciplinary panel of experts used a modified Delphi method to select drugs (or drug classes) with a potentially high risk-to-benefit ratio in older people and to reach consensus on their safest use, including explicit recommendations regarding drugs to avoid in all or selected circumstances. Aside from clinical use, the Beers Criteria have been used extensively in epidemiological studies, and implemented in healthcare settings to assess the quality of care [8, 15]. In the US, the National Committee for Quality Assurance (NCQA) has used the Beers Criteria as one of the main criteria to define the list of high risk medications (HRM) to avoid in the elderly. Based on the NCQA list, approximately a quarter of the US older population received at least one HRM in 2006 and 2007 [16, 17].

Estimating the burden of HRM, patterns, and appropriateness of their prescribing is essential to develop effective strategies for improving drug safety both in the UK and in other healthcare systems worldwide [18]. UK cross-sectional research [19, 20] has identified that increased number of drugs prescribed is consistently associated with greater exposure to potentially inappropriate prescribing; however it remains unknown whether the recent increases in prescribing rates are mirrored by a concomitant increase in harmful prescribing. Moreover, to the best of our knowledge, while exposure to at least one HRM (‘any exposure’) has always been the object of research, regular exposure to high risk medicines (‘long term exposure’) has not been investigated.

We investigated the current burden, trends and correlates of both any and long-term receipt of HRM using the 2012 Beers Criteria (adapted for the UK) in UK older patients in general practice. We also assessed the clinical conditions most frequently associated with HRM use and the appropriateness of HRM use according to Beers recommendations in 2011/12 patient records.

## Methods

### Cohort identification

We used a sample of patients registered with UK general practices contributing data to the Clinical Practice Research Datalink (CPRD) [21] GOLD version (available on July 2012). The protocol, including this and other research projects, was designed to investigate the health and prescribing burden of the oldest old over time in the UK by comparing this section of the population with younger age groups. The sampling procedure has been described elsewhere [22]. Briefly, to maximise the number of people with extreme ages in our dataset, we started by selecting centenarians registered at any time with general practices contributing to CPRD. Each centenarian (100 or over) was randomly matched (one to one for females, one to two for males) to younger patients aged 65 years or over, by practice, gender and calendar year in each of six younger age-groups (stratified by 5-10 year age bands) in order to have adequate number of younger patients in the control groups.

From the pool of 50,313 patients sampled, we initially drew a sub-sample of 21,377 patients including all subjects who turned 100 in the calendar years 2002/3, 2006/7, and 2010/11 and their matched pair. To account for potential end-of-life prescription changes, the 14,578 patients registered with the practice for the entire year were selected; thirty-one patients with uncertain date of death (discrepancy between CPRD and Office of National Statistics data) and 408 patients who died within three months after the end of each sampled year were also excluded. Finally, 239 patients with no clinical or therapy records at all for the three years prior to the sampled year were excluded as age-standardised rates of consulting behaviour in UK general practice [23] suggest that such individuals are ‘non-active’ (i.e. uncertain vital status).

Prescribing patterns were investigated for the three fiscal years (1st April through 31st March), noting that each subject contributed only to one of the three year groups. Due to the complexity of computing prescribing data, and for clarity of reporting we restricted our analysis to: 2011/12, the most recent year with complete data available; 2003/4, the year of publication of the previous revision of the Beers criteria [11] that coincided with widespread adoption of electronic medication coding in the UK; and 2007/8, an equidistant, intermediate time point between the previous two.

### Study measures

We extracted age categorised into age groups (65–84 years and 85+ years), sex (male, female), and as a proxy of socio-economic status we obtained quintiles of the Index of Multiple Deprivation (IMD, 2010) for England for those patients whose general practices had consented for patient postcodes to be linked to socio-economic data.

We included only prescription data for drugs with predominantly systemic effect (oral, sub-lingual, rectal, subcutaneous, intramuscular, intravenous route, or trans-dermal patch). Over-the-counter medicines were excluded as they are not routinely recorded in general practice records.

For each year, for each person we calculated the total number of drugs and HRM prescribed (i.e. at least once that year) to derive ‘any’ medication count. We then identified drugs or HRM that were prescribed for all quarters of the given year, as a proxy of ‘long-term’ prescribing.

We defined HRM using the 2012 Beers’ criteria, a list of 53 medications or medication classes potentially harmful in the older population [12]. We focused on the 34 drugs or drug classes defined as ‘drugs to avoid in older adults’. This group included a sub-group of drugs to be avoided only if an additional filtering condition (i.e. a Beers recommendation) was met (e.g. ‘alpha-blockers to be avoided only if used as anti-hypertensive drugs’). When analysing trends and main correlates, we reported the prevalence of HRM irrespective of whether the additional filter (if any) was present. To accommodate differences between the US and UK drug availability, two pharmacists and two geriatricians independently reviewed the drugs lists to identify modifications, in line with the British National Formulary [24]. The results were discussed and consensus reached: 47 drugs that are not available as UK-licensed prescription-only products were removed and the final list of 92 drugs (see Additional file 1) including five drugs substituted for their UK equivalents, and seven drugs (all benzodiazepines) added after checking whether any other extra substance (other than those included in the US) were included in the UK formulary (particularly historic preparations) (Additional file 1). Insulin was excluded because the sliding scale prescribing pattern could not be operationalised.

To document the appropriateness of their use in 2011/12, the additional filter (Beers recommendations) was applied to HRM prescribed to 1 % or more of the population. The methods used to operationalise the Beers recommendations are reported in Additional file 2.

For each year the prescribing burden of HRM (any or long-term) was assessed. To ensure adequate numbers contributed to analysis, we categorised measures of any HRM into four categories (none, one, two or three or more drugs) and long-term prescribing into three levels (none, one or two or more drugs). Drugs prevalence was assessed at the mid-point of the year 2011/12 (30^th^ September).

Multimorbidity was operationalised as the count of 15 chronic diseases included in previous research in UK general practice [25, 26]: hypertension, atrial fibrillation, coronary heart disease, chronic heart failure, stroke/transient ischaemic attack, cancer, chronic kidney disease [any stage], asthma, chronic obstructive pulmonary disease, dementia, depression, mental health disorders [psychoses, schizophrenia, bipolar affective disease], diabetes, hypothyroidism, and epilepsy).

Three common disabling conditions of ageing (osteoarthritis, osteoporosis and anaemia) were also categorised. The resultant multimorbidity index (range 0–18) was assessed at the mid-point of the fiscal year 2011/12 and further categorised (none, one to two, and three or more).

### Statistical analysis

To account for differences in population structure between 2003/4 and 2011/12 all estimates were weighted to the UK 2011 population composition (in five year age groups for men and women separately) based on the UK Office of National Statistics (ONS) data [27]. Data were reported as weighted percentages and variables compared using design-based chi-square tests.

Combining data from all three samples, we undertook multivariable logistic regression analyses (accounting for study design), to test the association between the outcome (i.e. any HRM exposure - none versus one or more HRM) and potential explanatory variables. Three models were developed for these analyses, adding progressively more covariates at each step (Model 1 = year sampled, age, gender; Model 2 = Model 1 plus medication count; and Model 3 = Model 2 plus multimorbidity and socio-economic status). The same modelling procedures were used for the modelling ‘long-term’ HRM (none versus one or more long-term HRM). Weighted and adjusted odds ratios and associated 95 % confidence intervals are presented for each model. Models were scrutinised for multicollinearity by reviewing the variance inflation factors. We also explored potential interactions between time (year sampled) and each explanatory variable on our outcome measures. Weighted and adjusted odds ratios and associated 95 % confidence intervals are presented for each model.

Additional analysis was undertaken in data restricted to the 2011/12 sample. Here we explored the associations between different medical conditions and HRM exposure (any or long-term), in multivariable models including dummy variables for the 18 individual chronic conditions and all other explanatory variables.

A conservative alpha level of 0.01 was chosen as the threshold for statistical significance.

Data were analysed using the Stata 13 (StataCorp. 2013. Stata Statistical Software: Release 13. College Station, TX: StataCorp LP).

### Ethics

The CPRD has been granted Multiple Research Ethics Committee approval (NRES Committee East Midlands – Derby, reference 05/MRE04/87) to undertake purely observational studies, with external data linkages including Hospital Episode Statistics and Office for National Statistics mortality data. The work of CPRD is also covered by NIGB-ECC approval ECC 5-05 (a) 2012. The present study was based on anonymised CPRD data used for observational research purposes. The study protocol was reviewed and approved by the Independent Scientific Advisory Committee (ISAC) for the UK Medicines and Healthcare products Regulatory Agency (protocol approved 12_017A3). While ISAC is primarily responsible for reviewing protocols for scientific quality, whenever study-specific ethical issues arise, it may recommend further ethical approval. In the case of this observational research no further ethics scrutiny was deemed necessary by ISAC.

## Results

The studied sample comprised 13,900 people registered with 504 UK general practices. Patients were broadly similar across time, in terms of age, gender and socio-economic status, although the proportions with no major chronic diseases decreased between 2003/4 and 2011/12 (Table 1).

**Table 1:** Characteristics of the study sample by year

	Fiscal year			
	2003/4	2007/8	2011/12	*p*-value
Sample size (unweighted)	3882	4756	5262	
	Weighted % (95 % CI)	Weighted % (95 % CI)	Weighted % (95 % CI)	
Age				0.862
65 to 84 years	86.6 (86.0, 87.2)	86.6 (86.0, 87.1)	86.4 (86.0, 86.8)	
85 years or over	13.4 (12.8, 14.0)	13.4 (12.9, 14.0)	13.6 (13.2, 14.0)	
Sex				0.997
Women	56.0 (50.9, 61.0)	55.8 (51.5, 59.9)	55.8 (51.4, 60.2)	
Socio-economic status (index of multiple deprivation quintile)				0.430
Least deprived quintile	15.9 (12.7, 19.7)	17.8 (14.3, 22.0)	20.0 (16.8, 23.6)	
2nd quintile	16.0 (13.5, 18.9)	18.7 (16.3, 21.3)	16.6 (14.4, 19.2)	
3rd quintile	14.2 (11.9, 16.7)	13.3 (11.1, 15.7)	13.2 (10.6, 16.4)	
4th quintile	12.6 (9.9, 15.9)	10.5 (8.3, 13.2)	9.4 (7.7, 11.4)	
Most deprived quintile	8.4 (6.2, 11.2)	8.2 (6.5, 10.3)	6.0 (4.6, 7.7)	
Missing (no linkage)	33.0 (27.4, 39.0)	31.6 (26.7, 37.0)	34.7 (29.3, 40.6)	
Multimorbidity (number of chronic conditions)				<0.001
0	21.9 (19.5, 24.6)	18.7 (16.9, 20.8)	16.6 (14.9, 18.3)	
1 to 2	50.1 (47.5, 52.8)	46.9 (44.5, 49.2)	46.4 (44.3, 48.5)	
3 or more	27.9 (25.8, 30.2)	34.4 (32.3, 36.6)	37.0 (35.0, 39.2)	
Medications count (number of drugs prescribed at least once during the year)				<0.001
0	12.2 (10.5, 14.0)	9.7 (8.4, 11.3)	7.8 (6.6, 9.2)	
1	8.7 (7.4, 10.2)	7.1 (6.0, 8.4)	5.9 (4.9, 7.0)	
2 to 4	26.9 (24.8, 29.2)	25.3 (23.4, 27.3)	25.7 (23.8, 27.6)	
5 to 9	35.8 (33.5, 38.0)	36.6 (34.7, 38.5)	36.1 (34.0, 38.2)	
10 or more	16.4 (14.7, 18.3)	21.2 (19.6, 23.0)	24.6 (22.8, 26.5)	
Medications count (number of drugs prescribed all quarters)				<0.001
0	33.9 (31.5, 36.4)	26.5 (24.5, 28.5)	21.7 (19.8, 23.8)	
1	14.0 (12.5, 15.8)	12.8 (11.5, 14.4)	12.3 (10.9, 13.7)	
2 to 4	35.6 (33.3, 37.9)	35.7 (33.9, 37.5)	37.1 (35.0, 39.2)	
5 to 9	15.1 (13.6, 16.8)	23.2 (21.6, 24.9)	25.2 (23.3, 27.0)	
10 or more	1.3 (0.8, 2.2)	1.8 (1.4, 2.4)	3.8 (3.1, 4.6)	

Medication counts significantly increased over time for any and long-term prescribing (Table 1). The proportion of patients receiving any ten or more drugs increased (16.4 % to 24.6 %; *p* < 0.001) and a similar pattern was observed for long-term prescribing (1.3 % to 3.8 %; *p* < 0.001). In contrast, the prevalence of any HRM remained stable over time (2003/4: 38.7 %, 95 % CI 36.4 to 41.1 %; 20007/8: 36.9 %, 34.9 % to 39.0 %; 2011/12: 38.4 %, 36.3 to 40.5; *p* = 0.468). The prevalence of long-term use of HRM was also stable (2003/4: 18.5 %, 95 % CI 16.8 to 20.3; 20007/8: 17.7 %, 16.3 % to 19.2 %; 2011/12: 17.4 %, 15.9 to 19.9; *p* = 0.609) accounting for approximately half of the any HRM prescribing burden. Additional file 3 summarises the percentages of people prescribed individual HRMs for each year.

After adjusting for age and gender (Table 2, Model 1), there was no difference in the risk of any HRM exposure among the three sample years. When the rising medication count was included (Model 2), the adjusted risk of HRM exposure was lower in 2007/08 and 2011/12 compared with 2003/04. Increased multimorbidity burden (Model 3) was protective, while female sex and higher medication counts conferred a greater risk of HRM. Oldest age was not associated with any HRM. Overall, similar results were found when we analysed the risk of long-term HRM (Table 3), with the notable exception of oldest age, which was now associated with greater risk of long-term HRM (OR: 1.16; 95 % CI 1.04, 1.28; *p* = 0.007). There was no evidence of multicollinearity in any models. No evidence of statistical interactions between the year sampled and other explanatory variables were observed, suggesting that the underlying associations between the correlates (sex, medication count, and multimorbidity) and HRM remained consistent across the different sampling years (data not presented).

**Table 2:** Logistic regression models for any HRM prescribing

	Model 1		Model 2		Model 3	
Sample size (unweighted)^a^	12,532		12,532		12,532	
	Weighted OR (95 % CI)	*p*-value	Weighted OR (95 % CI)	*p*-value	Weighted OR (95 % CI)	*p*-value
Year sampled						
2003/4	1		1		1	
2007/8	0.88 (0.76, 1.00)	0.059	0.77 (0.67, 0.89)	<0.001	0.78 (0.67, 0.90)	0.001
2011/12	0.91 (0.79, 1.03)	0.157	0.76 (0.65, 0.87)	<0.001	0.76 (0.65, 0.87)	<0.001
Age						
65 to 84 years	1		1		1	
85 years or over	1.06 (0.97, 1.16)	0.175	0.89 (0.81, 0.97)	0.012	0.90 (0.82, 0.99)	0.033
Sex						
Men	1		1		1	
Women	1.28 (1.13, 1.43)	<0.001	1.18 (1.04, 1.33)	0.007	1.18 (1.04, 1.33)	0.008
Medications count (number of drugs)						
1			1		1	
2 to 4			3.16 (2.22, 4.49)	<0.001	3.66 (2.57, 5.20)	<0.001
5 to 9			6.46 (4.54, 9.18)	<0.001	8.44 (5.92, 12.0)	<0.001
10 or more			18.23 (12.7, 26.0)	<0.001	25.46 (17.7, 36.5)	<0.001
Multimorbidity (number of diseases)						
None					1	
1 to 2					0.60 (0.49, 0.73)	<0.001
3 or more					0.54 (0.43, 0.67)	<0.001
Socio-economic status (IMD^b^ quintiles)						
Least deprived					1	
2nd					1.13 (0.92, 1.37)	0.231
3rd					0.97 (0.79, 1.17)	0.734
4th					0.97 (0.75, 1.23)	0.809
Most deprived					0.85 (0.65, 1.08)	0.189
Missing					1.03 (0.87, 1.21)	0.741

^a^Sample restricted to patients with at least 1 drug prescription. ^b^ *IMD* Index of Multiple Deprivation

**Table 3:** Logistic regression models for long-term HRM prescribing

	Model 1		Model 2		Model 3	
Sample size (unweighted)^a^	12,532		12,532		12,532	
	Weighted OR (95 % CI)	*p*-value	Weighted OR (95 % CI)	*p*-value	Weighted OR (95 % CI)	*p*-value
Year sampled						
2003/4	1		1		1	
2007/8	0.82 (0.70, 0.96)	0.015	0.73 (0.61, 0.85)	<0.001	0.73 (0.62, 0.86)	<0.001
2011/12	0.74 (0.63, 0.87)	0.000	0.61 (0.51, 0.71)	<0.001	0.61 (0.51, 0.72)	<0.001
Age						
65 to 84 years	1		1		1	
85 years or over	1.26 (1.13, 1.39)	0.000	1.15 (1.03, 1.28)	0.009	1.16 (1.04, 1.28)	0.007
Sex						
Men			1		1	
Women	1.35 (1.17, 1.56)	0.000	1.40 (1.20, 1.62)	<0.001	1.41 (1.20, 1.63)	<0.001
Medications count (number of drugs)						
1			1		1	
2 to 4			2.27 (1.75, 2.93)	<0.001	2.46 (1.91, 3.16)	<0.001
5 to 9			4.71 (3.62, 6.12)	<0.001	5.33 (4.08, 6.94)	<0.001
10 or more			15.44 (10.0, 23.7)	<0.001	17.77 (11.4, 27.5)	<0.001
None					1	
1 to 2					0.66 (0.49, 0.86)	0.003
3 or more					0.62 (0.46, 0.83)	0.001
Socio-economic status (IMD^b^ quintiles)						
Least deprived					1	
2nd					1.11 (0.87, 1.41)	0.402
3rd					1.02 (0.79, 1.30)	0.886
4th					1.08 (0.82, 1.43)	0.569
Most deprived					1.07 (0.79, 1.42)	0.660
Missing					0.99 (0.80, 1.22)	0.953

^a^Sample restricted to patients with at least 1 drug prescription. ^b^ *IMD* Index of Multiple Deprivation

We then restricted analysis to the most recent patient sample from 2011/12. Accounting for confounders including age, gender, number of drugs, Index of Multiple Deprivation and all individual chronic conditions included in the multimorbidity count (see above), patients with osteoarthritis were at higher risk of any HRM, while those with atrial fibrillation were at increased risk of long-term HRM (Additional file 4). Patients with cardiovascular risk factors or cardiovascular disease (diabetes, hypertension, coronary heart disease, and stroke/transient ischaemic attack) were at reduced risk of any or long-term HRM. (See Additional file 5 for the unadjusted weighted prevalence of any or long-term HRM for all 18 clinical conditions).

**Fig. 1:**
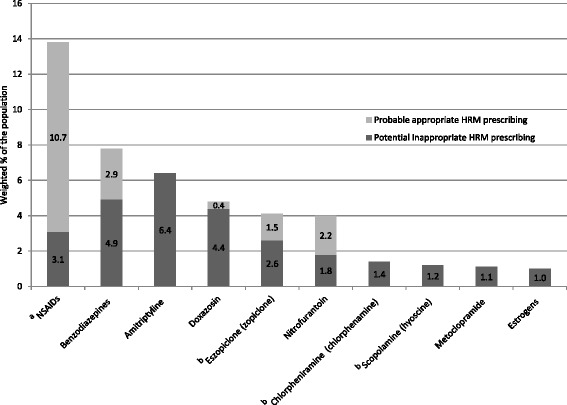
Potential inappropriate high risk medication prescribing. Legend to Fig. 1: Proportion of patients with potential inappropriate HRM prescribing (for drugs prescribed to at least 1 % of the sample) filtered using Beers recommendations. ^a^NSAIDs: non-steroidal anti-inflammatory drugs; ^b^UK equivalent reported in brackets

We then explored any HRM exposure that was prevalent in at least 1 % of the population (Fig. 1) in 2011/12. Overall, the ten drugs included in this subgroup accounted for 85 % of all patients prescribed a HRM. The latter included non-steroidal anti-inflammatory drugs (NSAIDs) (13.8 % of patients; mainly ibuprofen/naproxen), benzodiazepines (7.8 %; mostly temazepam/diazepam), amitriptyline (6.4 %), doxazosin (4.8 %), zopliclone (4.1 %), nitrofurantoin (4.0 %), chlorphenamine (1.4 %), hyoscine (1.2 %) metoclopramide (1.1 %) and oestrogens (1.0 %). Although the rates were lower, a similar pattern of use was observed for long-term HRM with the exception of nitrofurantoin whose prevalence was negligible (Additional file 3). When Beers recommendations were applied, amitriptyline emerged as the most prevalent potentially inappropriate medication (6.4 %), followed by benzodiazepines (4.9 %), doxazosin (4.4 %); the rate for NSAIDs had dropped considerably (3.1 %) (Fig. 1).

## Discussion

We present the first UK primary care data on the prevalence, trends over time, correlates and appropriateness of high risk medication prescribing in older people using the updated 2012 Beers Criteria adapted for the UK. The proportion of older people exposed to HRM remained constant across time against a background of increasing polypharmacy burden. While a third of older people are exposed to HRM, only half of them receive HRM prescriptions over the long-term. Drug burden and female sex were associated with any and long-term HRM, but oldest age (85 years or older) was only correlated with long-term HRM use. No association with socio-economic status was found. Patients with osteoarthritis and atrial fibrillation were at greater risk of HRM, while the presence of cardiovascular risk factors or cardiovascular disease was associated with lower risk. Ten drugs/drug classes accounted for almost all HRM prescribing in 2011/12.

Direct comparisons between our results and previous UK findings are also difficult due to differences in definitions and methodology. Our definition of HRM is conceptually similar with the Beers 2003 version [11] of “potentially inappropriate medications” (PIMs), where few explicit filtering recommendations were made. Three UK studies applying the 2003 Beers criteria to general practice records estimated the proportions of patients receiving any PIM at 32.2 % in 2003 [28], and 28.3 % [28] and 31.0 % [19] in 2005/6. These figures are similar, although somewhat lower than our estimates.

We found that patients sampled in later years were at a slightly reduced statistical risk of HRM exposure compared with people registered in 2003/4. The observed risk reduction may be due to concomitant increase in non-HRM drug prescribing rather than a change in HRM prescribing over time. Previous research has found that people aged 85 and older are less likely to receive one or more HRM per year [16, 29]. Similarly, we found that, accounting for confounders, older age (85 years and over) was associated with lower risk of ‘any’ HRM exposure, even though this result did not reach our pre-defined level of statistical significance (OR: 0.90, 95 % CI: 0.82, 0.99; *p* = 0.033). Conversely, the oldest old were at increased risk of continuous HRM exposure. The fact that people aged 85 and older, the most vulnerable section of the older population, are at greater risk of long-term HRM is somehow concerning. To the best of our knowledge this finding has not been previously reported and would merit further investigation. These data suggest that, in older people, shorter and long-term HRM exposure need to be investigated separately and interventions to reduce HRM may need to target both shorter and long-term use. Consistent with previous reports, we found being female, and taking increasing numbers of concomitant medications are strongly associated with the risk of PIMs exposure [19, 20]. While socio-economic status was found to be weakly associated in previous UK [19] and in the US reports [16], we observed no such effect.

Our finding that increased multimorbidity index was protective of HRM exposure requires careful consideration and might be the effect of confounding by indication. When the four cardiovascular conditions (hypertension, diabetes, coronary heart disease, stroke/transient ischaemic attack) were excluded, the index was no longer associated with HRM risk (data not shown). This may be due to potential confounding as QOF cardiovascular conditions may be subject to enhanced monitoring; in addition, NSAIDs and amitriptyline both have explicit notes regarding cautionary prescribing in patients with cardiovascular risk. Patients with osteoarthritis and atrial fibrillation were at increased HRM risk, largely due to NSAIDs and antiarrhythmic agents (digoxin, amiodarone and sotalol) prescribing (data not shown).

Our methodology has some limitations. While most chronic diseases have been shown to be reliably recorded in CPRD [30] some question its validity [31]. Prescribing data, the focus of this study, is deemed to be mostly accurate as the issuing of scripts is computerised [31]. However, we cannot completely eliminate the risk of limited under-recording since a small proportion of scripts may be handwritten. This might particularly affect the drugs prescribed during home visits (e.g. antibiotics, pain killers etc.). Another limitation is that we were unable to code residential status (even though the CPRD database includes people living in nursing and residential home), which was previously associated with PIMs use [19, 20]. As mentioned, due to the observational nature of the study residual confounding in some of the correlations found is also possible. Finally, due to the fact that only a subset of high risk medications has been considered, prescribing of HRM could be underestimated.

While the Beers list is extensive, our analysis illustrated that in the UK, HRM exposure was largely attributable to ten drugs/drug classes. This includes sedatives, non-steroidal anti-inflammatory drugs and anticholinergic drugs which are captured in other prescribing optimisation tools such as START/STOPP [32], and hence some of the identified modified Beers drugs are also prevalent historically in the international literature [15]. Reducing or optimising the use of the ten drugs/drug classes, particularly NSAIDs, benzodiazepines and amitriptyline, could dramatically reduce HRM in older patients, and particularly those belonging to high-risk groups. That HRM prescribing has not decreased over time requires careful consideration. The reality of clinical practice is that it may not always be feasible to avoid HRMs. As part of medical decision making, physicians may be driven to use higher risk drugs, making a careful trade-off between attempts to relieve symptoms directly affecting an older person’s quality of life, against the risk of future adverse consequences. Consistent with this, four of the ten prevalent HRMs we identified have strong sedative effects. Although UK insomnia management guidelines recommend cognitive and behavioural interventions [33], the treatment of sleep disorders, in cognitively intact and impaired people, might be one of the key drivers of HRM resulting from an attempt to manage this highly distressing condition.

## Conclusions

HRMs rates for older people from UK general practices participating to CPRD remain largely unchanged since 2003/4 against the backdrop of increasing polypharmacy. Future research is needed exploring why HRM prescribing remains entrenched especially in the oldest old and in women, and to support the development of targeted interventions to improve prescribing safety.

## Additional files

Additional file 1:
**High Risk Medications to avoid in older people according to Beers Criteria after UK adaptation.** (DOCX 43 kb) Additional file 2:
**Beers 2012 Criteria filtering conditions (recommendations) and their operationalization in the CPRD sample.** (DOCX 44 kb) Additional file 3:
**Prevalence of any and** **long-term** **high risk medications in 2003/4, 2007/8 and 2011/12.** (DOCX 59 kb) Additional file 4:
**Logistic regression models for the predictors of high risk medication exposure at least once a year (any prescribing) or all quarters of the year (long-term prescribing) in 2011/12.** (DOCX 43 kb) Additional file 5:
**Weighted prevalence of patients receiving one or more potentially harmful drugs in 2011/12 by specific chronic disease.** (DOCX 46 kb)

## Abbreviations

CI: confidence interval
CPRD: Clinical Practice Research Datalink
HRM: high risk medication
IMD: Index of Multiple Deprivation
MHRA: UK Medicines and Healthcare products Regulatory Agency
NCQA: US National Committee for Quality Assurance
NSAID: non-steroidal anti-inflammatory drug
OR: odds ratio
PIM: potentially inappropriate prescribing
QOF: UK Quality and Outcomes Framework
START/STOPP: Screening Tool to Alert doctors to Right i.e. appropriate, indicated Treatments. /Screening Tool of Older People’s potentially inappropriate Prescriptions
UK: United Kingdom
US: United States
