# Supplementary material for: Any versus long-term prescribing of high risk medications in older people using 2012 Beers Criteria: results from three cross-sectional samples of primary care records for 2003/4, 2007/8 and 2011/12
Source: BMC Geriatr. 2015 Nov 5;15:146. doi: 10.1186/s12877-015-0143-8 (PMC4635594; doi:10.1186/s12877-015-0143-8)
Supplement: Additional file 1: — High Risk Medications to avoid in older people according to Beers Criteria after UK adaptation. (DOCX 43 kb) [file 12877_2015_143_MOESM1_ESM.docx]

Additional file 1. High Risk Medications to avoid in older people according to 2012 Beers Criteria after UK adaptation

| **Anti-infective** | **Tricyclic ant-depressants** | **Antipsychotic drugs (second generation)** | **Other CNS** |
| --- | --- | --- | --- |
| Nitrofurantoin* | Amitriptyline | Aripiprazole* | Phenobarbital |
| **Gastrointestinal** | Clomipramine | Clozapine* | Chloral hydrate |
| Metoclopramide* | Doxepin | Olanzapine* | Meprobamate* |
| **Endocrine** | Imipramine | Paliperidone* | Dihydroergotamine |
| Glibenclamide (UK substitute for ‘glyburide’) | Trimipramine | Quetiapine* |  |
| Megestrol |  | Risperidone* | **NSAIDs** |
| Oestrogens* | **Antihistamines** |  | Aspirin (>325mg/day) |
| Testosterone* | Chlorphenamine (chlorpheniramine in US) | **Benzodiazepines (short- or intermediate acting)** | Diclofenac* |
| Somatropin* | Clemastine | Alprazolam* | Etodolac* |
|  | Cyproheptadine | Clobazam*∫ | Ibuprofen* |
| **Cardiovascular)** | Doxylamine | Ketazolam*∫ | Ketoprofen* |
| Methyldopa | Hydroxyzine | Lorazepam* | Mefenamic acid* |
| Nifedipine IR | Promethazine | Medazepam*∫ | Meloxicam* |
| Clonidine* | Triprolidine | Oxazepam* | Nabumetone* |
| Doxazosin* |  | Temazepam* | Naproxen* |
| Prazosin* | **Anti-Parkinson’s and muscle relaxants** | **Benzodiazepines (long- acting)** | Piroxicam* |
| Terazosin* | Trihexyphenidyl | Bromazepam*∫ | Sulindac* |
| Spironolactone(>25mg)* | Orphenadrine | Chlordiazepoxide* | Indomethacin |
| Dipyridamole IR | Methocarbamol | Clonazepam* | Ketorolac |
| Amiodarone* |  | Diazepam* |  |
| Dronedarone* | **Antipsychotic drugs (first generation)** | Flunitrazepam* |  |
| Flecainide* | Chlorpromazine* | Flurazepam* |  |
| Propafenone* | Fluphenazine* | Lormetazepam*∫ | **Other pain medications** |
| Sotalol* | Haloperidol* | Nitrazepam*∫ | Pethidine (UK substitute for ‘meperidine’) |
| Disopyramide | Perphenazine* | Triazolam*∫ | Pentazocine |
| Digoxin (>0.125mg) | Pimozide* |  |  |
| **Anti-spasmodics** | Levomepromazine* | **Non-benzodiazepine hypnotics** | ***Key*** |
| Dicycloverine (UK substitute for ‘dicyclomine’)* | Promazine* | Zopiclone (UK substitute for ‘eszopiclone’)* | *= Avoid if additional filtering conditions met |
| Propantheline* | Trifluoperazine* | Zolpidem* | ∫ = UK preparation not listed in Beers |
| Hyoscine (UK substitute for ‘scopolamine’)* |  | Zaleplon* |  |
