# Supplementary material for: Any versus long-term prescribing of high risk medications in older people using 2012 Beers Criteria: results from three cross-sectional samples of primary care records for 2003/4, 2007/8 and 2011/12
Source: BMC Geriatr. 2015 Nov 5;15:146. doi: 10.1186/s12877-015-0143-8 (PMC4635594; doi:10.1186/s12877-015-0143-8)
Supplement: Additional file 2: — Beers 2012 Criteria filtering conditions (recommendations) and their operationalization in the CPRD sample. (DOCX 44 kb) [file 12877_2015_143_MOESM2_ESM.docx]

Additional file 2: Beers 2012 Criteria filtering conditions (recommendations) and their operationalization in the CPRD sample

| **Potentially harmful drug** | **2012 Beers Criteria filtering condition (i.e. recommendation** [**^1^**](#_ENREF_1)**)** | **Operationalization of potentially inappropriate prescription using CPRD data** |
| --- | --- | --- |
| *Non-COX-selective NSAIDs* | Avoid chronic use unless other alternatives are not effective and patient can take gastroprotective agent (proton pump inhibitor or misoprostol) | Prescriptions in ≥ 2 quarters without concomitant prescription of proton pump inhibitor or misoprostol in the same quarter |
| *Benzodiazepines* | Avoid benzodiazepines (any type) for treatment of insomnia, agitation, or delirium. May be appropriate for seizure disorders, rapid eye movement sleep disorders, benzodiazepine withdrawal, ethanol withdrawal, severe generalized anxiety disorder, periprocedural anaesthesia, end-of-life care | No mention of seizure disorders, rapid eye movement sleep disorders, benzodiazepine withdrawal, ethanol withdrawal, or generalized anxiety disorder or equivalent wording in the patient’s clinical records. Periprocedural anaesthesia and end-of-life care excluded by the study design. |
| *Amitriptyline* | Avoid | No filtering condition (always potentially inappropriate) |
| *Doxazosin* | Avoid use as an antihypertensive | No mention of benign prostate hyperplasia or equivalent wording in the patient’s clinical records |
| *Eszopiclone (zopiclone)* | Avoid chronic use (> 90 days) | Prescriptions in ≥ 2 quarters |
| *Nitrofurantoin* | Avoid for long-term suppression; avoid in patients with creatinine clearance < 60 mL/min | Prescriptions in ≥ 2 quarters or in subjects with creatinine clearance < 60 mL/min |
| *Chlorpheniramine (chlorphenamine)* | Avoid | No filtering condition (always potentially inappropriate) |
| *Scopolamine (hyoscine)* | Avoid except in short-term palliative care to decrease oral secretions | No filtering condition (palliative care excluded by the study design) |
| *Metoclopramide* | Avoid, unless for gastroparesis | No mention of gastroparesis or similar wording in the patient’s clinical records |
| *Estrogens* | Avoid oral and topical patch | No filtering condition (preparations different from oral or topic patch were excluded by the study design) |
