# Supplementary material for: Any versus long-term prescribing of high risk medications in older people using 2012 Beers Criteria: results from three cross-sectional samples of primary care records for 2003/4, 2007/8 and 2011/12
Source: BMC Geriatr. 2015 Nov 5;15:146. doi: 10.1186/s12877-015-0143-8 (PMC4635594; doi:10.1186/s12877-015-0143-8)
Supplement: Additional file 3: — Prevalence of any and long-term high risk medications in 2003/4, 2007/8 and 2011/12. (DOCX 59 kb) [file 12877_2015_143_MOESM3_ESM.docx]

Additional file 3: Prevalence of any and long-term high risk medications (HRM) in 2003/4, 2007/8 and 2011/12

|  | **Any HRM** | | |  | **Long-term HRM** | | |  |
| --- | --- | --- | --- | --- | --- | --- | --- | --- |
|  | **2003/4** | **2007/8** | **2011/12** |  | **2003/4** | **2007/8** | **2011/12** |  |
| **Drug class or individual drugs ^a^** | **Weighted %**  **(95% CI)** | **Weighted %**  **(95% CI)** | **Weighted %**  **(95% CI)** | ***p-value*** | **Weighted %**  **(95% CI)** | **Weighted %**  **(95% CI)** | **Weighted %**  **(95% CI)** | ***p-value*** |
| **Anti-infective** |  |  |  |  |  |  |  |  |
| Nitrofurantoin | 1 (0.7, 1.5) | 1.8 (1.4, 2.5) | 4 (3.4, 4.8) | <0.001 | 0 (0.0, 0.1) | 0 (0.0, 0.1) | 0.3 (0.2, 0.5) | <0.001 |
| **Gastrointestinal** |  |  |  |  |  |  |  |  |
| Metoclopramide | 1.3 (0.9, 1.9) | 1.3 (0.9, 1.9) | 1.1 (0.8, 1.5) | 0.762 | 0.3 (0.1, 0.6) | 0.1 (0.0, 0.3) | 0.2 (0.1, 0.3) | 0.276 |
| **Endocrine** |  |  |  |  |  |  |  |  |
| Glyburide (glibenclamide) | 0.4 (0.1, 1.0) | 0.2 (0.1, 0.4) | 0.1 (0.0, 0.2) | 0.061 | 0.2 (0.1, 0.8) | 0.1 (0.0, 0.2) | 0 (0.0, 0.2) | 0.102 |
| Megestrol | 0 (0.0, 0.0) | 0 (0.0, 0.2) | 0 (0.0, 0.0) | 0.019 | 0 | 0 (0.0, 0.2) | 0 (0.0, 0.0) | 0.189 |
| Estrogens | 2.8 (2.1, 3.6) | 1.2 (0.8, 1.8) | 1 (0.7, 1.5) | <0.001 | 0.7 (0.4, 1.2) | 0.5 (0.3, 0.9) | 0.5 (0.3, 0.8) | 0.528 |
| Somatropin | 0 | 0 | 0 (0.0, 0.1) | 0.765 | 0 | 0 | 0 (0.0, 0.1) | 0.765 |
| **Antispasmodics** |  |  |  |  |  |  |  |  |
| Dicyclomine (dicycloverine) | 0.2 (0.1, 0.5) | 0.2 (0.1, 0.5) | 0.1 (0.0, 0.2) | 0.284 | 0 (0.0, 0.1) | 0.1 (0.0, 0.2) | 0 (0.0, 0.1) | 0.444 |
| Propantheline | 0 | 0 (0.0, 0.1) | 0 | 0.727 | 0 | 0 (0.0, 0.1) | 0 | 0.728 |
| Scopolamine (hyoscine) | 0.1 (0.0, 0.3) | 0.5 (0.3, 0.9) | 1.2 (0.9, 1.8) | <0.001 | 0 (0.0, 0.1) | 0.1 (0.0, 0.3) | 0.2 (0.1, 0.6) | 0.059 |
| **Cardiovascular** | | | | | | | | |
| Methyldopa | 0.1 (0.1, 0.3) | 0.1 (0.0, 0.3) | 0 (0.0, 0.2) | 0.453 | 0.1 (0.0, 0.2) | 0.1 (0.0, 0.2) | 0 (0.0, 0.2) | 0.871 |
| Nifedipine (IR) | 2.3 (1.8, 3.1) | 1.4 (1.0, 1.8) | 0.6 (0.4, 0.9) | <0.001 | 1.6 (1.1, 2.3) | 1.1 (0.8, 1.6) | 0.2 (0.1, 0.4) | <0.001 |
| Clonidine | 0.1 (0.0, 0.3) | 0.2 (0.1, 0.5) | 0.2 (0.1, 0.5) | 0.445 | 0 (0.0, 0.1) | 0.1 (0.0, 0.4) | 0.1 (0.0, 0.3) | 0.434 |
| Doxasozin | 4.4 (3.5, 5.5) | 4.5 (3.7, 5.4) | 4.8 (3.9, 5.9) | 0.790 | 2.9 (2.2, 3.7) | 3.3 (2.7, 4.1) | 3.8 (3.0, 4.7) | 0.279 |
| Prazosin | 0.2 (0.1, 0.8) | 0.1 (0.0, 0.2) | 0.1 (0.0, 0.2) | 0.157 | 0.2 (0.0, 0.8) | 0 (0.0, 0.2) | 0.1 (0.0, 0.2) | 0.202 |
| Terazosin | 0.4 (0.2, 1.0) | 0.1 (0.0, 0.2) | 0.2 (0.1, 0.7) | 0.083 | 0.4 (0.2, 1.0) | 0 (0.0, 0.2) | 0.2 (0.1, 0.7) | 0.053 |
| Spironolactone (>25 mg) | 0.5 (0.3, 0.9) | 0.2 (0.1, 0.3) | 0.5 (0.3, 0.9) | 0.048 | 0.3 (0.1, 0.7) | 0.1 (0.0, 0.2) | 0.2 (0.1, 0.4) | 0.095 |
| Dipyridamole (IR) | 0.2 (0.1, 0.5) | 0.2 (0.1, 0.3) | 0.1 (0.0, 0.1) | 0.178 | 0.1 (0.0, 0.3) | 0.1 (0.0, 0.2) | 0 (0.0, 0.1) | 0.623 |
| Digoxin (>0.125 mg) | 1.4 (0.9, 2.2) | 1.1 (0.7, 1.7) | 0.6 (0.4, 0.9) | 0.051 | 0.8 (0.5, 1.5) | 0.9 (0.6, 1.5) | 0.6 (0.4, 0.8) | 0.370 |
| Disopyramide | 0.1 (0.0, 0.2) | 0 (0.0, 0.0) | 0.1 (0.0, 0.2) | 0.158 | 0.1 (0.0, 0.2) | 0 (0.0, 0.0) | 0.1 (0.0, 0.2) | 0.167 |
| Amiodarone | 1.5 (1.0, 2.3) | 0.9 (0.6, 1.2) | 0.7 (0.4, 1.0) | 0.010 | 1.1 (0.7, 1.9) | 0.7 (0.5, 1.0) | 0.6 (0.4, 0.9) | 0.095 |
| Dronedarone | 0 | 0 | 0.1 (0.0, 0.3) | 0.400 | 0 | 0 | 0.1 (0.0, 0.3) | 0.400 |
| Flecainide | 0.3 (0.1, 1.0) | 0.5 (0.3, 0.9) | 0.2 (0.1, 0.4) | 0.350 | 0.2 (0.0, 0.8) | 0.5 (0.3, 0.8) | 0.1 (0.0, 0.2) | 0.083 |
| Propafenone | 0 (0.0, 0.0) | 0 (0.0, 0.1) | 0 (0.0, 0.1) | 0.426 | 0 (0.0, 0.0) | 0 (0.0, 0.1) | 0 (0.0, 0.1) | 0.453 |
| Sotalol | 0.9 (0.5, 1.5) | 0.9 (0.6, 1.3) | 0.7 (0.4, 1.0) | 0.571 | 0.5 (0.3, 0.8) | 0.7 (0.5, 1.0) | 0.5 (0.3, 0.9) | 0.442 |
| **Tertiary TCAs** |  |  |  |  |  |  |  |  |
| Amitriptyline | 4.2 (3.3, 5.2) | 4.5 (3.8, 5.5) | 6.4 (5.4, 7.6) | 0.002 | 1.5 (1.1, 2.2) | 1.8 (1.3, 2.4) | 2.7 (2.1, 3.4) | 0.014 |
| Clomipramine | 0.4 (0.2, 0.9) | 0.2 (0.1, 0.7) | 0.1 (0.0, 0.2) | 0.102 | 0.2 (0.1, 0.5) | 0.1 (0.0, 0.3) | 0 (0.0, 0.1) | 0.133 |
| Doxepin | 0.1 (0.0, 0.2) | 0.1 (0.0, 0.2) | 0 (0.0, 0.2) | 0.604 | 0 (0.0, 0.2) | 0.1 (0.0, 0.2) | 0 (0.0, 0.1) | 0.640 |
| Imipramine | 0.2 (0.1, 0.4) | 0.2 (0.1, 0.7) | 0.1 (0.0, 0.1) | 0.354 | 0.1 (0.0, 0.4) | 0 (0.0, 0.1) | 0 (0.0, 0.1) | 0.363 |
| Trimipramine | 0 (0.0, 0.1) | 0.2 (0.1, 0.5) | 0 (0.0, 0.1) | 0.002 | 0 | 0.1 (0.0, 0.3) | 0 (0.0, 0.1) | 0.122 |
| **Anti-histamines (1^st^ generation)** |  |  |  |  |  |  |  |  |
| Chlorpheniramine (chlorphenamine) | 1.1 (0.7, 1.6) | 1 (0.7, 1.3) | 1.4 (1.0, 2.0) | 0.284 | 0.1 (0.0, 0.2) | 0.1 (0.0, 0.2) | 0.2 (0.1, 0.5) | 0.104 |
| Clemastine | 0.1 (0.0, 0.3) | 0 (0.0, 0.0) | 0 | 0.060 | 0 (0.0, 0.3) | 0 (0.0, 0.0) | 0 | 0.161 |
| Cyproheptadine | 0 (0.0, 0.1) | 0 | 0 | 0.701 | 0 | 0 | 0 |  |
| Hydroxyzine | 0.4 (0.3, 0.8) | 0.4 (0.2, 0.7) | 0.3 (0.2, 0.5) | 0.730 | 0 (0.0, 0.1) | 0.1 (0.0, 0.4) | 0 (0.0, 0.1) | 0.047 |
| Promethazine | 0 (0.0, 0.1) | 0.2 (0.1, 0.4) | 0.4 (0.2, 0.8) | 0.011 | 0 (0.0, 0.0) | 0 (0.0, 0.1) | 0.1 (0.0, 0.3) | 0.001 |
| **Anti-Parkinson’s agents & muscle relaxants** | | | | | | | | |
| Trihexyphenidyl | 0.2 (0.0, 0.9) | 0 | 0.1 (0.0, 0.4) | 0.390 | 0.1 (0.0, 0.9) | 0 | 0.1 (0.0, 0.4) | 0.449 |
| Orphenadrine | 0.1 (0.0, 0.2) | 0 (0.0, 0.1) | 0.1 (0.0, 0.2) | 0.645 | 0.1 (0.0, 0.2) | 0 | 0.1 (0.0, 0.2) | 0.404 |
| Methocarbamol | 0.1 (0.0, 0.2) | 0 (0.0, 0.2) | 0.1 (0.0, 0.4) | 0.546 | 0 | 0 | 0.1 (0.0, 0.4) | 0.605 |
| **Other CNS drugs** |  |  |  |  |  |  |  |  |
| Phenobarbital | 0.2 (0.1, 0.4) | 0.2 (0.1, 0.7) | 0.3 (0.1, 0.7) | 0.677 | 0.1 (0.1, 0.3) | 0.2 (0.1, 0.7) | 0.3 (0.1, 0.7) | 0.485 |
| Chloral hydrate | 0 | 0 | 0 (0.0, 0.0) | 0.902 | 0 | 0 | 0 |  |
| Meprobamate | 0 (0.0, 0.3) | 0 (0.0, 0.0) | 0 | 0.158 | 0 | 0 (0.0, 0.0) | 0 | 0.961 |
| **Antipsychotics (1^st^ generation)** |  |  |  |  |  |  |  |  |
| Chlorpromazine | 0.2 (0.1, 0.3) | 0.2 (0.1, 0.5) | 0 (0.0, 0.1) | 0.014 | 0.1 (0.1, 0.3) | 0.2 (0.1, 0.4) | 0 (0.0, 0.1) | 0.044 |
| Fluphenazine | 0.1 (0.0, 0.4) | 0 (0.0, 0.2) | 0 | 0.297 | 0.1 (0.0, 0.4) | 0 | 0 | 0.480 |
| Haloperidol | 0.1 (0.0, 0.2) | 0.1 (0.0, 0.2) | 0.2 (0.1, 0.3) | 0.585 | 0 (0.0, 0.0) | 0.1 (0.0, 0.1) | 0.1 (0.0, 0.3) | 0.053 |
| Perphenazine | 0.1 (0.0, 0.2) | 0.1 (0.0, 0.3) | 0.1 (0.0, 0.5) | 0.961 | 0.1 (0.0, 0.2) | 0.1 (0.0, 0.3) | 0 | 0.363 |
| Levomepromazine | 0.1 (0.0, 0.4) | 0 | 0 (0.0, 0.1) | 0.108 | 0 (0.0, 0.3) | 0 | 0 | 0.605 |
| Promazine | 0 (0.0, 0.1) | 0.1 (0.0, 0.2) | 0.3 (0.1, 0.8) | <0.001 | 0 (0.0, 0.0) | 0 (0.0, 0.1) | 0.1 (0.0, 0.1) | 0.032 |
| Trifluoperazine | 0.2 (0.1, 0.4) | 0 (0.0, 0.1) | 0.2 (0.1, 0.4) | 0.102 | 0.1 (0.0, 0.3) | 0 (0.0, 0.1) | 0.1 (0.0, 0.3) | 0.279 |
| **Antipsychotics (2^nd^ generation)** |  |  |  |  |  |  |  |  |
| Aripiprazole | 0 | 0 (0.0, 0.1) | 0.1 (0.0, 0.2) | 0.118 | 0 | 0 | 0 (0.0, 0.1) | 0.615 |
| Olanzapine | 0.4 (0.2, 0.8) | 0.3 (0.1, 0.5) | 0.6 (0.4, 1.1) | 0.113 | 0.3 (0.1, 0.7) | 0.2 (0.1, 0.4) | 0.4 (0.2, 0.9) | 0.343 |
| Quetiapine | 0.1 (0.0, 0.3) | 0.6 (0.4, 0.8) | 0.6 (0.4, 0.9) | <0.001 | 0.1 (0.0, 0.2) | 0.3 (0.2, 0.5) | 0.4 (0.2, 0.7) | 0.005 |
| Risperidone | 0.7 (0.5, 1.0) | 0.4 (0.2, 0.8) | 0.2 (0.1, 0.4) | 0.028 | 0.3 (0.2, 0.4) | 0.3 (0.1, 0.7) | 0.1 (0.0, 0.3) | 0.336 |
| **Benzodiazepines** | **8.7 (7.4, 10.2)** | **7.2 (6.3, 8.2)** | **7.8 (6.8, 8.8)** | **0.172** | **4.4 (3.6, 5.4)** | **3.7 (3.1, 4.4)** | **3.4 (2.8, 4.2)** | **0.168** |
| *Short- & intermediate-acting* | *4.5 (3.7, 5.5)* | *3.6 (3.0, 4.4)* | *3.9 (3.3, 4.7)* | *0.269* | *2.5 (1.9, 3.2)* | *1.9 (1.5, 2.4)* | *1.9 (1.5, 2.4)* | *0.193* |
| Alprazolam | 0 | 0 (0.0, 0.3) | 0 (0.0, 0.1) | 0.468 | 0 | 0 (0.0, 0.3) | 0 (0.0, 0.1) | 0.469 |
| Lorazepam | 0.6 (0.4, 0.9) | 0.5 (0.3, 0.8) | 0.8 (0.5, 1.1) | 0.409 | 0.3 (0.2, 0.6) | 0.2 (0.1, 0.4) | 0.3 (0.2, 0.5) | 0.602 |
| Oxazepam | 0.4 (0.2, 0.8) | 0.1 (0.0, 0.1) | 0.2 (0.1, 0.5) | 0.028 | 0.1 (0.0, 0.3) | 0 (0.0, 0.1) | 0.1 (0.0, 0.4) | 0.397 |
| Temazepam | 3.6 (2.8, 4.6) | 3 (2.4, 3.7) | 3 (2.5, 3.8) | 0.433 | 2.1 (1.5, 2.7) | 1.6 (1.3, 2.0) | 1.5 (1.1, 2.0) | 0.194 |
| Clobazam | 0 (0.0, 0.2) | 0 (0.0, 0.3) | 0 | 0.536 | 0 | 0 (0.0, 0.3) | 0 | 0.640 |
| *Long-acting* | *4.5 (3.7, 5.6)* | *4 (3.3, 4.7)* | *4.2 (3.6, 5.1)* | *0.609* | *2 (1.5, 2.6)* | *1.8 (1.4, 2.4)* | *1.5 (1.1, 2.0)* | *0.362* |
| Chlordiazepoxide | 0.2 (0.1, 0.5) | 0.2 (0.1, 0.4) | 0.1 (0.1, 0.3) | 0.893 | 0.1 (0.0, 0.3) | 0.1 (0.0, 0.4) | 0.1 (0.0, 0.2) | 0.608 |
| Clonazepam | 0.2 (0.1, 0.4) | 0.3 (0.1, 0.6) | 0.3 (0.1, 0.6) | 0.637 | 0.1 (0.0, 0.3) | 0.1 (0.1, 0.4) | 0.2 (0.1, 0.5) | 0.662 |
| Diazepam | 2.6 (2.0, 3.4) | 2.7 (2.2, 3.4) | 3.4 (2.8, 4.2) | 0.177 | 0.8 (0.5, 1.2) | 0.9 (0.6, 1.3) | 0.9 (0.6, 1.4) | 0.834 |
| Flurazepam | 0.1 (0.0, 0.3) | 0 (0.0, 0.1) | 0 | 0.049 | 0.1 (0.0, 0.3) | 0 (0.0, 0.1) | 0 | 0.132 |
| Lormetazepam | 0.3 (0.1, 0.6) | 0.3 (0.2, 0.5) | 0 (0.0, 0.2) | 0.037 | 0.1 (0.0, 0.2) | 0.1 (0.0, 0.2) | 0 (0.0, 0.1) | 0.121 |
| Nitrazepam | 1.4 (1.0, 1.9) | 0.8 (0.5, 1.1) | 0.4 (0.3, 0.6) | <0.001 | 0.9 (0.6, 1.4) | 0.6 (0.4, 0.8) | 0.3 (0.2, 0.4) | 0.001 |
| **Non-benzodiazepine hypnotics** |  |  |  |  |  |  |  |  |
| Eszopiclone (zopiclone) | 2.8 (2.2, 3.6) | 2.5 (2.0, 3.1) | 4.1 (3.3, 5.0) | 0.003 | 0.8 (0.5, 1.3) | 1 (0.7, 1.4) | 1.4 (1.1, 1.9) | 0.080 |
| Zolpidem | 0.5 (0.2, 1.2) | 0.4 (0.2, 0.8) | 0.3 (0.2, 0.6) | 0.611 | 0.1 (0.0, 0.2) | 0.3 (0.1, 0.7) | 0.2 (0.1, 0.4) | 0.139 |
| Zaleplon | 0.1 (0.0, 0.3) | 0 (0.0, 0.2) | 0 | 0.346 | 0 (0.0, 0.1) | 0 | 0 | 0.707 |
| **NSAIDs** | **13.3 (11.7, 15.1)** | **15.2 (13.7, 16.9)** | **13.8 (12.4, 15.3)** | **0.236** | **3.1 (2.4, 4.1)** | **3.2 (2.5, 4.0)** | **3.1 (2.4, 4.0)** | **0.996** |
| Diclofenac | 4.9 (3.9, 6.2) | 6.4 (5.4, 7.7) | 3.5 (2.7, 4.5) | <0.001 | 0.7 (0.5, 1.2) | 1 (0.6, 1.6) | 0.6 (0.4, 1.1) | 0.440 |
| Etodolac | 0.3 (0.1, 0.6) | 0.3 (0.1, 0.6) | 0.1 (0.1, 0.3) | 0.395 | 0.1 (0.0, 0.4) | 0.2 (0.1, 0.6) | 0.1 (0.0, 0.3) | 0.249 |
| Ibuprofen | 5.3 (4.3, 6.5) | 5.8 (5.0, 6.8) | 5.1 (4.2, 6.1) | 0.568 | 1.2 (0.7, 1.9) | 0.7 (0.5, 1.2) | 0.7 (0.4, 1.1) | 0.154 |
| Ketoprofen | 0.1 (0.0, 0.3) | 0.1 (0.0, 0.3) | 0.1 (0.0, 0.3) | 0.981 | 0.1 (0.0, 0.3) | 0 (0.0, 0.1) | 0 (0.0, 0.2) | 0.426 |
| Mefenamic acid | 0 (0.0, 0.1) | 0.1 (0.0, 0.3) | 0 (0.0, 0.3) | 0.507 | 0 | 0 | 0 |  |
| Meloxicam | 1.3 (0.9, 1.9) | 1.3 (0.9, 1.9) | 0.7 (0.4, 1.1) | 0.055 | 0.3 (0.2, 0.7) | 0.6 (0.3, 1.1) | 0.5 (0.3, 0.9) | 0.537 |
| Nabumetone | 0.2 (0.1, 0.5) | 0.2 (0.1, 0.4) | 0 (0.0, 0.2) | 0.184 | 0 (0.0, 0.2) | 0 (0.0, 0.1) | 0 (0.0, 0.2) | 0.956 |
| Naproxen | 1 (0.7, 1.6) | 1.8 (1.3, 2.5) | 5.7 (4.7, 6.8) | <0.001 | 0.2 (0.1, 0.5) | 0.3 (0.1, 0.6) | 0.5 (0.3, 1.0) | 0.214 |
| Piroxicam | 0.5 (0.2, 1.0) | 0.4 (0.1, 0.9) | 0 (0.0, 0.2) | 0.031 | 0.2 (0.0, 0.8) | 0.1 (0.0, 0.2) | 0 | 0.295 |
| Sulindac | 0 (0.0, 0.1) | 0 (0.0, 0.1) | 0 | 0.527 | 0 (0.0, 0.1) | 0 | 0 | 0.708 |
| Indomethacin | 0.7 (0.3, 1.3) | 0.4 (0.2, 0.9) | 0.3 (0.2, 0.7) | 0.387 | 0.2 (0.0, 0.8) | 0.1 (0.0, 0.3) | 0.1 (0.0, 0.4) | 0.712 |
| **Other pain relief** |  |  |  |  |  |  |  |  |
| Pentazocine | 0 (0.0, 0.0) | 0 | 0 | 0.807 | 0 (0.0, 0.0) | 0 | 0 | 0.808 |

***** Fifteen drugs were identified as eligible, but were not found in any records during electronic searching of the fiscal years: testosterone (endocrine); doxylamine and triprolidine (antihistamines); PHPozide (1^st^ generation antipsychotic); clozapine and paliperidone (2^nd^ generation antipsychotics); ketazolam and medazepam (short- & intermediate acting benzodiazepines); bromazepam, flunitrazepam and triazolam (long-acting benzodiazepines); dihydroergotamine (other CNS drug); aspirin (>325 mg) and ketorolac (NSAIDs); and meperidine (pethidine, other pain relief).

CNS: Central nervous system; IR: immediate release; NSAIDs: Non-steroidal anti-inflammatory drugs; TCA: Tricyclic antidepressant
