# Supplementary material for: Any versus long-term prescribing of high risk medications in older people using 2012 Beers Criteria: results from three cross-sectional samples of primary care records for 2003/4, 2007/8 and 2011/12
Source: BMC Geriatr. 2015 Nov 5;15:146. doi: 10.1186/s12877-015-0143-8 (PMC4635594; doi:10.1186/s12877-015-0143-8)
Supplement: Additional file 4: — Logistic regression models for the predictors of high risk medication exposure at least once a year (any prescribing) or all quarters of the year (long-term prescribing) in 2011/12. (DOCX 43 kb) [file 12877_2015_143_MOESM4_ESM.docx]

Additional file 4: Logistic regression models for the predictors of HRM exposure at least once a year (any prescribing) or for all quarters of the year (long-term prescribing) in 2011/12

|  | **Any PHP** | | **Sustained PHP** | |
| --- | --- | --- | --- | --- |
| Sample size (unweighted) | 4909* | | 4301* | |
|  | Weighted OR* (95% CI) | p-value | Weighted OR* (95% CI) | p-value |
| *Disease* |  |  |  |  |
| No Disease (Ref.) | 1 |  | 1 |  |
| Hypertension | 0.80 (0.66 to 0.96) | 0.018 | 0.74 (0.60 to 0.92) | 0.007 |
| Diabetes | 0.66 (0.49 to 0.88) | 0.005 | 0.68 (0.49 to 0.92) | 0.014 |
| Stroke/TIA | 0.74 (0.55 to 0.99) | 0.048 | 0.63 (0.45 to 0.87) | 0.006 |
| CHD | 0.51 (0.39 to 0.66) | <0.001 | 0.53 (0.38 to 0.72) | <0.001 |
| Atrial fibrillation | 0.89 (0.67 to 1.16) | 0.391 | 1.57 (1.14 to 2.15) | 0.006 |
| Chronic heart failure | 0.97 (0.66 to 1.41) | 0.873 | 0.78 (0.51 to 1.19) | 0.261 |
| Asthma | 1.18 (0.86 to 1.61) | 0.289 | 1.44 (1.00 to 2.06) | 0.045 |
| COPD | 0.75 (0.54 to 1.03) | 0.078 | 0.77 (0.52 to 1.12) | 0.182 |
| CKD | 1.01 (0.82 to 1.24) | 0.895 | 0.93 (0.71 to 1.19) | 0.567 |
| Cancer | 0.78 (0.58 to 1.05) | 0.108 | 0.82 (0.55 to 1.20) | 0.308 |
| Dementia | 1.10 (0.74 to 1.62) | 0.625 | 0.98 (0.61 to 1.56) | 0.948 |
| Depression | 1.29 (0.99 to 1.67) | 0.058 | 1.34 (0.97 to 1.82) | 0.069 |
| MHD | 2.73 (1.19 to 6.24) | 0.017 | 2.80 (1.12 to 6.94) | 0.027 |
| Epilepsy | 3.02 (1.15 to 7.91) | 0.024 | 2.20 (0.91 to 5.29) | 0.079 |
| Hypothyroidism | 1.00 (0.74 to 1.34) | 0.984 | 0.83 (0.59 to 1.14) | 0.246 |
| Anaemia | 0.79 (0.60 to 1.01) | 0.069 | 0.74 (0.53 to 1.04) | 0.085 |
| Osteoarthritis | 1.32 (1.07 to 1.62) | 0.007 | 1.12 (0.87 to 1.41) | 0.365 |
| Osteoporosis | 0.87 (0.67 to 1.13) | 0.302 | 0.79 (0.57 to 1.08) | 0.149 |

Sample restricted to patients receiving at least 1 prescription.

*Model including age, gender, number of drugs, Index of Multiple Deprivation and all diseases.

CHD: Coronary Heart Disease; COPD: Chronic Obstructive Pulmonary Disease; CKD: Chronic Kidney Disease (any stage); MHD: Mental health disorders= psychoses, schizophrenia, bipolar affective disorder; HRM: High Risk Medications; TIA: transient ischaemic attack.
