# Supplementary material for: Any versus long-term prescribing of high risk medications in older people using 2012 Beers Criteria: results from three cross-sectional samples of primary care records for 2003/4, 2007/8 and 2011/12
Source: BMC Geriatr. 2015 Nov 5;15:146. doi: 10.1186/s12877-015-0143-8 (PMC4635594; doi:10.1186/s12877-015-0143-8)
Supplement: Additional file 5: — Weighted prevalence of patients receiving one or more potentially harmful drugs in 2011/12 by specific chronic disease. (DOCX 46 kb) [file 12877_2015_143_MOESM5_ESM.docx]

Additional file 5: Weighted prevalence of patients receiving one or more high risk medications (HRM) in 2011/12 by specific chronic disease

|  | **Any HRM** | | |  | **Long-term HRM** | | |
| --- | --- | --- | --- | --- | --- | --- | --- |
| Sample size n (unweighted) | 5,262 | |  |  | 5,262 | |  |
|  | Weighted % (95% CI) | Weighted % (95% CI) |  |  | Weighted % (95% CI) | Weighted % (95% CI) |  |
| ***Disease*** | **Diagnosis absent** | **Diagnosis present** | p-value |  | **Diagnosis absent** | **Diagnosis present** | p-value |
| Hypertension | 35.6 (33.0 to 38.3) | 41.9 (38.9 to 45.0) | 0.002 |  | 15.3 (13.4 to 17.4) | 20 (17.9 to 22.3) | 0.001 |
| Diabetes | 37.8 (35.6 to 40.0) | 42.3 (36.4 to 48.5) | 0.161 |  | 16.5 (15.0 to 18.2) | 22.9 (18.8 to 27.6) | 0.004 |
| Stroke/TIA | 37.8 (35.6 to 40.1) | 44.5 (38.6 to 50.6) | 0.041 |  | 17.1 (15.5 to 18.8) | 20.2 (16.2 to 25.0) | 0.186 |
| CHD | 37.4 (35.2 to 39.6) | 43.6 (38.7 to 48.7) | 0.020 |  | 16.3 (14.8 to 18.0) | 23.0 (19.1 to 27.4) | 0.001 |
| Atrial fibrillation | 37.6 (35.3 to 39.9) | 47.7 (42.3 to 53.2) | 0.001 |  | 16.2 (14.7 to 17.8) | 31.3 (26.5 to 36.6) | <0.001 |
| Chronic heart failure | 38.0 (35.8 to 40.2) | 50.0 (41.4 to 58.6) | 0.008 |  | 17.0 (15.5 to 18.6) | 27.8 (21.6 to 35.0) | <0.001 |
| Asthma | 37.6 (35.3 to 39.9) | 47.7 (42.3 to 53.2) | 0.002 |  | 16.6 (15.1 to 18.2) | 25.3 (19.8 to 31.7) | 0.002 |
| COPD | 37.8 (35.7 to 40.0) | 45.2 (38.1 to 52.6) | 0.047 |  | 17.2 (15.7 to 18.8) | 20.2 (15.4 to 25.9) | 0.252 |
| CKD | 36.3 (33.9 to 38.7) | 45.6 (41.8 to 49.5) | <0.001 |  | 15.8 (14.1 to 17.6) | 22.9 (20.0 to 26.1) | <0.001 |
| Cancer | 38.3 (36.1 to 40.6) | 39.2 (33.2 to 45.6) | 0.785 |  | 17.4 (15.9 to 19.1) | 17.0 (12.6 to 22.5) | 0.873 |
| Dementia | 38.0 (35.9 to 40.2) | 48.8 (40.3 to 57.3) | 0.014 |  | 17.2 (15.7 to 18.8) | 22.9 (16.7 to 30.4) | 0.075 |
| Depression | 36.3 (34.1 to 38.5) | 51.8 (46.0 to 57.7) | <0.001 |  | 16 (14.5 to 17.6) | 26.4 (21.7 to 31.7) | <0.001 |
| Mental Health Disorder | 38.0 (35.9 to 40.1) | 71.0 (54.8 to 83.2) | <0.001 |  | 17.0 (15.5 to 18.6) | 46.5 (30.3 to 63.5) | <0.001 |
| Epilepsy | 38 (35.9 to 40.1) | 71 (48.6 to 86.4) | 0.002 |  | 17 (15.6 to 18.6) | 47.5 (27.6 to 68.1) | <0.001 |
| Hypothyroidism | 37.3 (35.1 to 39.6) | 49.3 (42.8 to 55.9) | 0.001 |  | 16.7 (15.2 to 18.3) | 24.3 (19.1 to 30.4) | 0.005 |
| Anaemia | 37.8 (35.6 to 40.0) | 48.4 (42.2 to 54.6) | 0.001 |  | 17.1 (15.5 to 18.7) | 22.7 (17.8 to 28.4) | 0.035 |
| Osteoarthritis | 33.8 (31.5 to 36.1) | 48.8 (45.1 to 52.6) | <0.001 |  | 15.4 (13.8 to 17.2) | 21.8 (18.9 to 24.9) | <0.001 |
| Osteoporosis | 37.2 (35.0 to 39.5) | 49.3 (43.8 to 54.8) | <0.001 |  | 16.7 (15.2 to 18.3) | 23.4 (19.2 to 28.2) | 0.002 |

CHD: Coronary Heart Disease; COPD: Chronic Obstructive Pulmonary Disease; CKD: Chronic Kidney Disease (any stage); MHD: Mental health disorders= psychoses, schizophrenia, bipolar affective disorder; TIA: transient ischaemic attack.
